# Therapeutic Effects and Mechanisms of Herbal Medicines for Treating Polycystic Ovary Syndrome: A Review

**DOI:** 10.3389/fphar.2020.01192

**Published:** 2020-08-12

**Authors:** Chan-Young Kwon, Ik-Hyun Cho, Kyoung Sun Park

**Affiliations:** ^1^ Department of Oriental Neuropsychiatry, Dong-eui University College of Korean Medicine, Busan, South Korea; ^2^ Department of Convergence Medical Science, Brain Korea 21 Plus Program, and Institute of Korean Medicine, College of Korean Medicine, Kyung Hee University, Seoul, South Korea; ^3^ Jaseng Hospital of Korean Medicine, Seoul, South Korea; ^4^ Jaseng Spine and Joint Research Institute, Jaseng Medical Foundation, Seoul, South Korea

**Keywords:** polycystic ovary syndrome, review, inflammation, oxidative stress, autophagy, apoptosis, nerve growth factor

## Abstract

**Background:**

Polycystic ovary syndrome (PCOS) is one of the most common disorders of endocrinology in reproductive-age women. In this study, we reviewed data on the effects and underlying mechanisms of herbal medicines used in the treatment of PCOS in laboratory studies.

**Methods:**

Articles published in English up to June 30, 2018 were searched in Medline and EMBASE. We extracted data regarding herbal intervention; target cell (or animal model) usage; method of herbal extraction; route of administration; dosage and periods; and outcomes of the compounds isolated from herbs, individual herbal extracts, and herbal formula decoctions. We summarized the actions and the mechanisms underlying the beneficial effects of herbal medicines on PCOS.

**Results:**

A total of 27 studies involving 22 herbal medicines reported their efficacy on PCOS. The herbal interventions in the 27 studies comprised four compounds isolated from herbs (6 studies), nine individual herbal extracts (11 studies), and nine herbal formula decoctions (10 studies). Herbal medicines normalized female hormones, diminished male hormones, recovered the estrous cycle, ameliorated insulin resistance, and improved lipid metabolism in PCOS. The mechanisms underlying the beneficial effects of herbal medicines on PCOS were found to be associated with anti-inflammation, anti-oxidative stress, inhibition of autophagy and/or apoptosis, and ovarian nerve growth factor reduction.

**Conclusions:**

Herbal medicines are thought to be promising resources in the development of effective therapeutic agents for PCOS. Further studies that include methodological quality assessment and quantitative synthesis of outcomes are recommended.

## Introduction

Polycystic ovary syndrome (PCOS) is one of the most common disorders of endocrinology in women of reproductive age. PCOS is diagnosed by confirming the presence of two of the following: oligo- and/or anovulation, clinical and/or biochemical hyperandrogenism, and ultrasound features of polycystic ovaries, with the exclusion of other etiologies (Fr and Tarlatzis, 2004). The prevalence rates of PCOS depend on the diagnostic criteria used, but they can be up to 18% when using the Rotterdam diagnostic criteria (Rotterdam ESHRE/ASRM-Sponsored PCOS Consensus Workshop Group, 2004). Hyperandrogenism is found in 60–80% of women with PCOS (Azziz et al., 2006). The major clinical or biochemical features of hyperandrogenism are acne, hirsutism, alopecia, and seborrheic dermatitis; elevated testosterone, androstenedione, and dehydroepiandrosterone sulfate levels; and decreased sex hormone binding globulin (SHBG) levels. The syndrome not only presents with reproductive manifestations but also has metabolic implications including insulin resistance, dyslipidemia, obesity, type 2 diabetes, and systemic inflammation (Deugarte et al., 2005; Sartor and Dickey, 2005; Escobar-Morreale et al., 2011).

While the first-line treatment for ovulation induction in women with PCOS is clomiphene citrate administration, the antiestrogenic effects of clomiphene citrate on the endometrium and cervical mucus are thought to cause a low conception rate of 20% (Gonen and Casper, 1990). Clomiphene citrate is also associated with a number of side effects including hot flushes, breast discomfort, abdominal distention, nausea, vomiting, nervousness, headache, hair loss, and disturbed vision (Legro et al., 2007a). Recent studies have investigated the role of metformin as an insulin-sensitizing agent, and although its use is increasing, the understanding of its mechanism is incomplete (Legro et al., 2007b). Moreover, it can cause the development of multiple follicles, along with a risk of ovarian hyperstimulation, multiple pregnancies, and congenital malformations such as neural tube defects, thereby leading to potentially unsatisfactory treatment outcomes.

Since PCOS is defined as a multifactorial metabolic-endocrine disorder (Laven et al., 2002), lifestyle and diet, and the Mediterranean diet in particular, play a relevant role, alongside pharmacological treatment (Barrea et al., 2019). Recent studies have suggested that complementary and alternative treatments, including herbal medicines and acupuncture, may alleviate PCOS symptoms, but evidence of their efficacy and safety is insufficient. Therefore, novel treatment strategies incorporating complementary and alternative therapies need to be investigated to optimize the treatment of PCOS. In this study, we reviewed data on the effects and underlying mechanisms of herbal medicines used in the treatment of PCOS model in laboratory studies.

## Methods

Articles published in English up to June 30, 2018 were searched in Medline (via PubMed) and EMBASE (via Elsevier). The search terms were a combination of medical subject heading (MeSH) terms and their synonyms. The search terms used were as follows: [herb* (Title/Abstract) OR Chinese herbal medicine (Title/Abstract) OR Chinese traditional medicine (MeSH) OR Korean medicine (Title/Abstract) OR Kampo medicine (MeSH)] AND [polycystic ovary syndrome (MeSH) OR polycystic ovarian syndrome (Title/Abstract)].

The inclusion criteria of our review included the following:

1. *In vitro* and *in vivo* studies that assessed the potential effects of herbal medicines on PCOS model
2. Research on the compounds isolated from herbs, individual herbal extracts, or herbal formula decoctions
3. Articles written in English

The exclusion criteria were as follows:

1. Clinical trials of herbal medicines for PCOS
2. Review articles
3. Articles that did not describe the components of the herbal medicine; however, this was allowed in the case of patented herbal medicines

Among the retrieved studies, after removing the duplicates, the titles and abstracts were reviewed to find potentially relevant articles. Then, the full-texts of screened articles were reviewed to confirm that they met our inclusion criteria.

We extracted data regarding herbal intervention; target cell (or animal model) usage; method of herbal extraction: route of administration; dosage and periods; and outcomes of the compounds isolated from herbs, individual herbal extracts, and herbal formula decoctions. Based on those data, we summarized the actions and the mechanisms underlying the beneficial effects of herbal medicines on PCOS model.

## Results

### Study Characteristics

**Figure 1:**
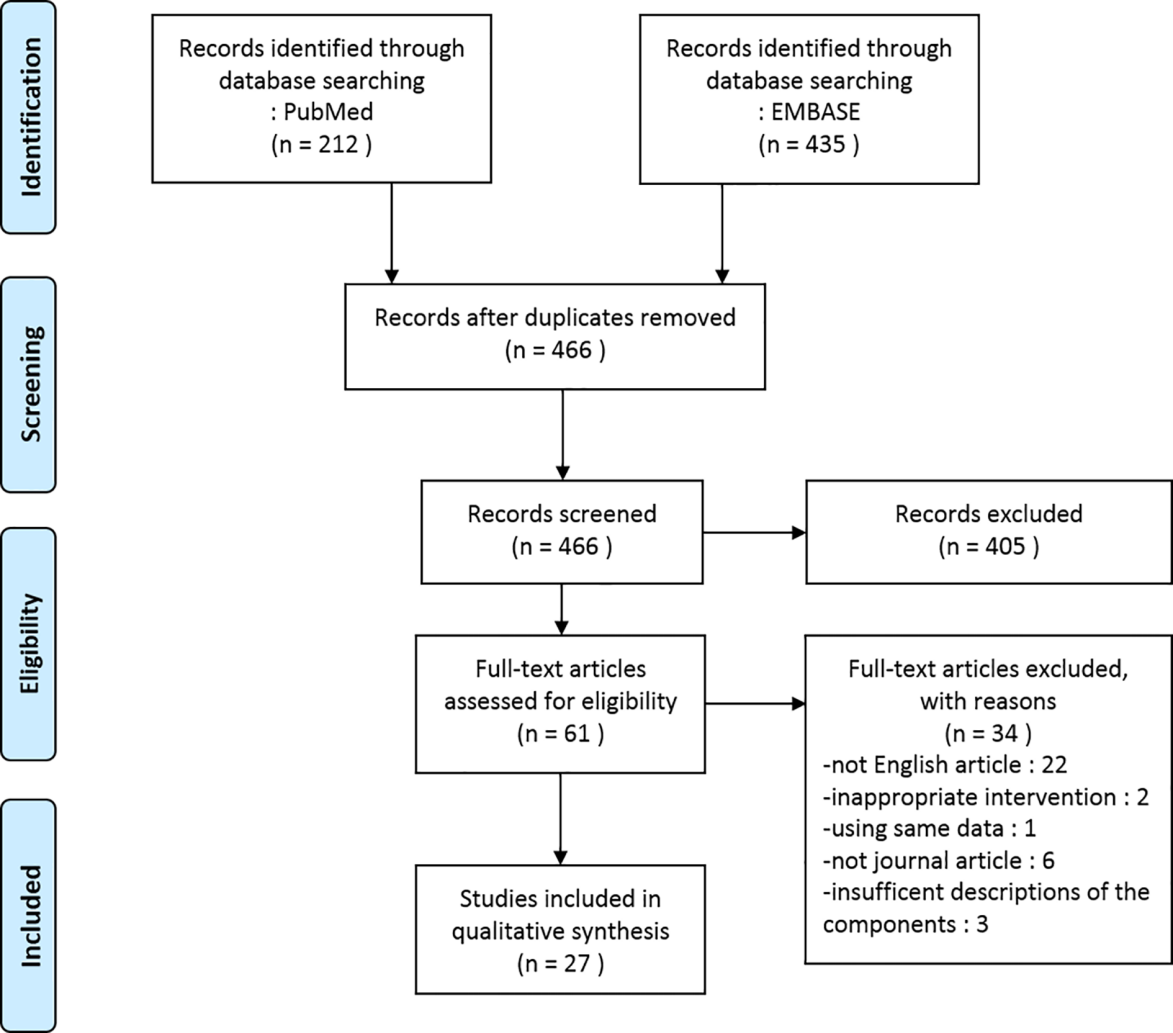
Flow diagram of the study selection process.

In the present review, we included a total of 27 studies involving 22 herbal medicines that reported their efficacy on PCOS model. We identified two *in vitro* studies, 22 *in vivo* studies, and three studies with both *in vitro* and *in vivo* experiments. The herbal interventions in the 27 studies comprised four compounds isolated from herbs (6 studies), nine individual herbal extracts (11 studies), and nine herbal formula decoctions (10 studies). A flow diagram of the article selection process is shown in **Figure 1**.

### Herbal Interventions and Their Laboratory Outcomes

#### Compounds Isolated From Herbs

Three studies revealed the effects of Cryptotanshinone: it reduced ovarian weight and body weight (Yang et al., 2011; Yu et al., 2014; Xia et al., 2017), the level of luteinising hormone (LH) and the LH/follicle-stimulating hormone (FSH) ratio (Yu et al., 2014; Xia et al., 2017), and the serum insulin and glucose levels (Yang et al., 2011; Yu et al., 2014). Iridoid (genipin, geniposide, and geniposidic acid) reduced the messenger RNA (mRNA) expressions of interleukin (IL)-1β, IL-6, IL-10, and inducible nitric oxide synthase (iNOS), and the over-secretion of nitrite (Zuo et al., 2017). Total saponins from Korean red ginseng (Panax ginseng C. A. Meyer) reduced the number of cystic follicles and the protein expression of nerve growth factor (NGF) in ovaries of Sprague Dawley (SD) female rats (Pak et al., 2005). Quercetin-treated female Wistar rats showed decreased levels of IL-1β, IL-6, and tumor necrosis factor (TNF)- α, and decreased insulin resistance (Wang et al., 2017). The data are summarized in **Table 1**.

**Table 1:** *In vitro* and *in vivo* studies of compounds isolated from herbs (n=6).

Interventions	Target cells or animal models	Herbal extraction	Route of administration	Dosage	Results	Author (year)
Cryptotanshinone	<*in vivo*>^(1)^ Adult SD female rats (aged 21 days, 35–40 g)	Purity > 98%	Oral	27 mg/kg/d For 3 weeks	1) Ovarian weight ↓, body weight ↓ 2) Ovarian quotiety ↓ 3) Normalization of estrous cycle 4) P (-), T ↓, E2 ↓ 5) LH ↓, FSH ↑, LH/FSH ratio ↓ 4) Activin A ↑, inhibin B ↓, follistatin ↓ 5) mRNA expressions of inhibin B and follistatin in ovaries ↓, mRNA expression of activin A in ovaries ↑ 6) Protein expressions of inhibin B and follistatin in ovaries ↓, protein expression of activin A in ovaries ↑	Xia et al. (2017)
	<*in vivo*>^(1)^ SPF grade female Wistar rats (aged 21 days, 35–40 g)	Purity = 99%	Oral	0.027 mg/g/d For 4 weeks	1) Recovery of estrous cycle 2) Body weight ↓ 3) Ovaries quotiety (= ovarian weight/body weight) ↓ 4) T ↓, androstenedione ↓, E2 (-), LH ↓, FSH (-), LH/FSH ↓, SHBG ↑ 5) TC (-), TG (-), LDL-C ↓, HDL-C (-), FPG (-), fasting insulin ↓ 6) Morphology: recovery of the color of ovarian tissue and number of granulosa cell layer, number of cystic follicles ↓, number of corpora lutea ↑ 7) mRNA expression of CYP17 in ovaries ↓, mRNA expressions of CYP19, AR, iGF-1, 3β-HSD and GDF-9 in ovaries (-) 8) Protein expressions of CYP17 and AR in ovaries ↓, protein expressions of CYP19, IGF-1, 3β-HSD and GDF-9 in ovaries (-)	Yu et al. (2014)
	<*in vivo*>^(2)^ Adult Wistar female rats (aged 12–14 weeks, 250–300 g)	Purity = 98%	Oral	0.027 mg/g/d For 14 days	1) Body weight ↓, ovarian weight ↓ 2) Improved OGTT profile 3) Serum insulin ↓, glucose ↓, insulin resistance ↓ 4) Recovery of estrous cycle 5) Number of atretic follicles ↓, number of corpora lutea ↑ 5) Serum levels of FSH, LH, and E2 (-), serum levels of 17-OH, androstenedione, and T ↓ 6) Protein expressions of IRS-1, IRS-2, PI3K p85α, and GLUT4 in ovaries ↑ 7) Protein expressions of ERK and CYP17 in ovaries ↓	Yang et al. (2011)
Iridoid (genipin, geniposide, and geniposidic acid)	<*in vitro*> RAW 264.7 and KGN cell lines	Purity ≥ 98%	–	1) genipin: 10, 50, 100 μM 2) geniposide: 10, 50, 100 μM 3) geinposidic acid: 10, 50, 100 μM	1) mRNA expression of IL-1β, IL-6, IL-10 and iNOS ↓ 2) Over-secretion of nitrite ↓ 3) Phosphorylation and degradation of IκB ↓ 4) NF-κB P65 ↑ 5) Nuclear entry of NF-κB P65 ↓ 6) Scavenge O2- (only genipin) 7) SOD activity ↓, HO-1 mRNA expression ↓, catalase activity ↑ 8) MiR-15b expression ↓	Zuo et al. (2017)
Korean red ginseng total saponins	<*in vivo*>^(3)^ SD female rats (190–210 g)	not reported	Intraperitoneally	100 mg/kg/2d For 60 days	1) Morphology: numbers of corpora lutea and corpora albicantia ↑, the number of cystic follicles ↓, the number of growing secondary follicle ↑ 2) Protein expression of NGF in ovaries ↓, protein expression of NGF in pituitary and hippocampus (-) 3) Ovarian weight ↓, body weight (-)	Pak et al. (2005)
Quercetin	<*in vivo*>^(1)^ Female Wistar rats (aged 21 days)	not reported	Gavage	100 mg/kg/d For 28 days	1) Recovery of estrous cycle 2) Body weight ↓ 3) Ovarian weight (-), ovary coefficients (-) 4) Morphology: follicles at various stages and more lutea, increased layers of granulosa cells within the follicle 5) Insulin ↓, IAUC ↓, FBG ↓ 6) Insulin resistance ↓ 7) IL-1β ↓, IL-6 ↓, TNF- α ↓ 8) Phosphorylation of IRS-1 ↑ 9) Nuclear translocation of NF-κB P65 ↓ 10) mRNA expressions of p22phox, OX-LDL, and TLR-4 in ovaries ↓ 11) Protein expressions of p22phox, OX-LDL, and TLR-4 in ovaries ↓	Wang et al. (2017)

17-OH, 17-Hydroxyprogesterone; AR, Androgen receptor; CYP17, Cytochrome P450 17alpha hydroxylase/17,20 lyase; CYP19, Cytochrome P450 aromatase; E2, Estradiol; ERK, Extracellular signal–regulated kinase; FBG, Fasting blood glucose; FPG, Fasting plasma glucose; FSH, Follicle-stimulating hormone; GDF-9, Growth differentiation factor-9; GULT4, Glucose transporter type 4; HDL-C, High density lipoprotein cholesterol; HO-1, Heme oxygenase-1; HSD, Hydroxysteroid dehydrogenase; IAUC, Area under the curve of blood insulin release; IGF-1, Insulin-like growth factor 1; IL, Interleukin; iNOS, Inducible nitric oxide synthase; IRS, Insulin receptor substrate; LDL-C, Low density lipoprotein cholesterol; LH, Luteinising hormone; NF-κB, Nuclear factor kappa B; NGF, Nerve growth factor; OGTT, Oral glucose tolerance test; OX-LDL, Oxidized low-density lipoprotein; P, Progesterone; PI3K, Phosphatidylinositol 3-kinase; SD, Sprague-Dawley; SHBG, Sex hormone-binding globulin; SOD, Superoxide dismutase; SPF, Specific-pathogen-free; T, Testosterone; TC, Total cholesterol; TG, Triglyceride; TLR, Toll-like receptor; TNF, Tumor necrosis factor.

In vivo polycystic ovary model by using ^(1)^ dehydroepiandrosterone; ^(2)^ testosterone propionate; ^(3)^ estradiol valerate; ^(4)^ letrozole; ^(5)^ dihydrotestosterone; ^(6)^ sodium prasterone sulfate; and ^(7)^ chronic unpredictable mild stress.

#### Individual Herbal Extracts

Aloe vera (L.) Burm.f. reduced plasma levels of total cholesterol (TC), triglyceride (TG), and low density lipoprotein cholesterol (LDL-C); enhanced high density lipoprotein cholesterol (HDL-C) levels (Desai et al., 2012); normalized follicular growth; and recovered the estrous cycle (Maharjan et al., 2010). Atractylodes macrocephala Koidz. induced the recovery of the estrous cycle and the reduction of testosterone levels, androstenedione levels, the free androgen index, LH levels, the LH/FSH ratio, and anti-Müllerian hormone levels (Zhou et al., 2016). Matricaria chamomilla L.-treated female Wistar rats showed decreased cysts in ovarian tissue and an increased number of dominant follicles (Zangeneh et al., 2010). Cocos nucifera L. recovered the estrous cycle; reduced TC, very low density cholesterol and TG levels; and increased HDL-C levels (Soumya et al., 2014). Linum usitatissimum L.-treated female SD rats showed increased antral follicles and corpus luteum, a decreased number of cystic follicles, and reduced diameter of antral follicles (Jelodar et al., 2018). Foeniculum vulgare Mill. induced normal glomerulus, normal basement membrane, and capillaries (Sadrefozalayi and Farokhi, 2014). Zingiber officinale Roscoe lowered the levels of LH and estrogen, and increased the levels of FSH and progesterone in neonatal female SD rats (Atashpour et al., 2017). Korean red ginseng (Panax ginseng C.A.Mey.)-treated SD female rats showed fewer cystic follicles and mRNA expression of NGF in ovaries (Pak et al., 2009; Jung et al., 2011). Labisia pumila var. alata enhanced the glucose infusion rate and reduced TC and TG levels (Mannerås et al., 2010). The data are summarized in **Table 2**.

**Table 2:** *In vitro* and *in vivo* studies of individual herbal extracts (n=11).

Interventions	Target cells or animal models	Herbal extraction	Route of administration	Dosage	Results	Author (year)
Aloe vera gel	<*in vivo*>^(4)^ Adult virgin female Charles Foster rats (aged 3–4 months, 200–225 g)	not reported	Oral	1 ml (10 mg)/d For 30 days	1) Ovarian weight (-) 2) 3β HSD activity ↓, 17β HSD activity (-) 3) Improved OGTT profile 4) Body weight ↓ 5) Plasma levels of TC ↓, TG ↓, HDL-C ↑, and LDL-C ↓ 6) Liver cholesterol ↓ 7) Activity of HMG-CoA reductase ↓ 8) Activity of LCAT (-)	Desai et al. (2012)
	<*in vivo*>^(4)^ [1] Adult virgin Charles Foster female rats (200–225 g) <*in vitro*> [2] Ovarian protein (30–35 g)	not reported	Oral	[1] 1 ml (10 mg)/d For 45 days [2] 1 mg For 1 h	[1] 1) Body weight ↓, ovarian weight ↓ 2) Recovery of estrous cycle 3) Improved OGTT profile 4) Histology: ovary atretic cysts ↓, normalization of follicular growth 5) Activities of ovarian 3β HSD and 17β HSD ↓ 6) Serum glutamate pyruvate (-), transaminase (-), creatinine (-) [2] 1) Activities of ovarian 3β HSD and 17β HSD ↓	Maharjan et al. (2010)
Atractylodes macrocephala koidz	<*in vivo*>^(2)^ [1] Female SD rats <*in vitro*> [2] Human ovarian granulosa-like KGN cells	Solvent: ethanol (70%) Time: three times for 0.5 h	Oral	[1] 0.1, 0.3, 0.9 g/kg/d For 8 weeks [2] 50, 200, 800 μg/mL For 24 h	[1] 1) Recovery of estrous cycle 2) Body weight ↓(only 0.3, 0.9 g/kg/d), ovarian weight ↓(only 0.9 g/kg/d), uterus weight (-) 3) T ↓, SHBG (-), free androgen index ↓, androstenedione ↓, LH ↓(only 0.3, 0.9 g/kg/d), FSH ↑, LH/FSH ↓, AMH ↓ 4) mRNA expression of FSHR in ovaries ↓, mRNA expression of AQP-9 in ovaries ↑ 5) Protein expression of FSHR in ovaries ↓, protein expression of AQP-9 in ovaries ↑ 6) ALT (-), AST (-), GGT (-) [2] 1) Protein expression of FSHR ↓, protein expression of AQP-9 ↑	Zhou et al. (2016)
Chamomile	<*in vivo*>^(3)^ Virgin adult cycling Wistar rats (aged 8 weeks, 200–220 g)	Solvent: ethanol (70%)	Intraperitoneally	25, 50, 75 mg/kg/d For 10 days	1) Morphology: cysts in ovarian tissue ↓(only 50 mg/kg/d), number of dominant follicles ↑(only 50 mg/kg/d), better endometrial tissue arrangements (only 50 mg/kg/d) 2) Serum levels of E2, LH, and FSH ↓	Zangeneh et al. (2010)
Cocus nucifera flower	<*in vivo*>^(4)^ Wistar female rats (100–150 g)	Solvent: aqueous alchhol (75%) Time: 12 h	Oral	100, 200 mg/kg/d For 4 weeks	1) Recovery of estrous cycle 2) Blood sugar level ↓ 3) TC ↓, VLDL-C ↓, HDL-C ↑, TG ↓ 4) Uterus weight ↑, ovary weight ↓ 5) Histophthology: number and diameter of cysts ↓, normal developing primary follicles ↑(only 200 mg/kg/d)	Soumya et al. (2014)
Flaxseed	<*in vivo*>^(3)^ Adult female SD rats (200 ± 20 g)	Solvent: ethanol (70%)	Gavage	200 mg/kg/d For 30 days	1) P ↑, T ↓, E2 (-), dehydroepiandrosterone (-) 2) Histomorphometric study: number of preantral follicles, antral follicles and corpora lutea ↑, number of cystic follicles and diameter of antral follicles ↓, number of primary follicle (-), thickness of granulosa layer ↑, thickness of theca layer and tunica albuginea ↓	Jelodar et al. (2018)
Foeniculum vulgare seeds	<*in vivo*>^(3)^ Adult female Wistar rats (200 ± 20 g)	Solvent: 400 ml of distilled water Time: 24 h	Gavage	100, 150 mg/kg/d For 4 weeks	1) Serum creatinine (-), serum urea ↓(only 150 mg/kg/d) 2) Histopathology: normal glomerulus, normal basement membrane, and capillaries; Bowman’s space (urinary space) and acute tubular necrosis were improved towards normal	Sadrefozalayi and Farokhi (2014)
Ginger	<*in vivo*>^(3)^ Neonatal female SD rats (aged 7–8 weeks, 170–200 g)	Solvent: ethanol (70%) Time: 48 h	Oral	4, 350 mg/kg/d For 88 weeks	1) LH ↓, FSH ↑, E ↓, P ↑	Atashpour et al. (2017)
Korean red ginseng	<*in vivo*>^(3)^ SD female rats (190–210 g)	not reported	Oral	200 mg/kg/d For 60 days	1) Histology: numbers of corpora lutea and corpora albicantia ↑, number of cystic follicles ↓ 2) mRNA expression of NGF in ovaries ↓, protein expression of NGF in ovaries ↓	Jung et al. (2011)
	<*in vivo*>^(3)^ SD female rats (190-210 g)	not reported	Oral	200 mg/kg/d For 60 days	1) Histology: numbers of corpora lutea and corpora albicantia ↑, number of cystic follicles ↓ 2) mRNA expression of NGF in ovaries ↓, protein expression of NGF in ovaries ↓	Pak et al. (2009)
Labisia pumila var. alata	<*in vivo*>^(5)^ Wistar female rats (aged 21 days)	Solvent: hot distilled water (80˚C) Time: 3 h	Oral	50 mg/kg/d For 4–5 weeks	1) Body weight (-), body composition (-) 2) Uterine weight ↑, weights of the inguinal, parametrial, retroperitoneal and mesenteric adipose tissue depots (-), weights of different hind limb muscles and the liver (-) 3) Size of adipocytes in mesenteric adipose tissue (-) 4) Glucose infusion rate ↑, plasma glucose (-) 5) TC ↓, TG ↓, HDL-C (-), LDL-C (-), resistin ↑, adiponectin (-), leptin (-) 6) mRNA expression of leptin in mesenteric adipose tissue ↓, mRNA expressions of resistin and adiponectin in mesenteric adipose tissue (-)	Mannerås et al. (2010)

ALT, Alanine aminotransferase; AMH, Anti-Müllerian hormone; AQP, Aquaporin; AST, Aspartate aminotransferase; E, Estrogen; FSH, Follicle-stimulating hormone; FSHR, Follicle-stimulating hormone receptor; GGT, Gamma glutamyltransferase; HDL-C, High density lipoprotein cholesterol; HMG-CoA, β-Hydroxy β-methylglutaryl-CoA; HSD, Hydroxysteroid dehydrogenase; LCAT, Lecithin–cholesterol acyltransferase; LDL-C, Low density lipoprotein cholesterol; LH, Luteinising hormone; NGF, Nerve growth factor; OGTT, Oral glucose tolerance test; P, Progesterone; SD, Sprague-Dawley; SHBG, Sex hormone-binding globulin; T, Testosterone; TC, Total cholesterol; TG, Triglyceride; VLDL-C, Very low density cholesterol.

In vivo polycystic ovary model by using ^(1)^ dehydroepiandrosterone; ^(2)^ testosterone propionate; ^(3)^ estradiol valerate; ^(4)^ letrozole; ^(5)^ dihydrotestosterone; ^(6)^ sodium prasterone sulfate; and ^(7)^ chronic unpredictable mild stress.

#### Herbal Formula Decoctions

Bushen Tongmai recipe (composed of Astragalus membranaceus Bge, Radix Polygoni Multiflori, Herba Cistanches, Radix Salviae Miltiorrhizae, Radix Notoginseng, Radix Puerariae, Herba Epimedii, Rhizoma Chuanxiong, and Radix Rehmanniae) reduced serum fasting insulin and enhanced protein expression of Protein kinase Bα in thecal and granulosa cells of antral follicles (Li et al., 2010). SD rats treated with Changbudodam-Tang (Rhizoma atractylodis, Rhizoma cyperi, Fructus ponciri, Pericarpium citri nobilis, Poria, Arisaematis Rhizoma, Radix glycyrrhizae, Massa Medicata Fermentata, and Zingiberis Rhizoma) and Yongdamsagan-Tang (Radix gentianae, Bupleuri Radix, Alimatis Rhizoma, Lignum akebiae, Plantaginis Semen, Hoelen, Rehmanniae Radix, Angelicae Gigantis Radix, Gardeniae Fructus, Scutellariae Radix, and Radix glycyrrhizae) showed a reduced number of cystic follicles, a higher number of growing secondary follicles, and reduced expression of NGF in ovaries (Lee et al., 2003). Chinese herbal medicine (CHM) 1 (Radix Astragali, Radix Rehmanniae Preparata, Cuscuta chinensis Lam, Fructus Ligustri Lucidi, Fructus Psoraleae, Radix Salviae Miltiorrhizae, and Rubus idaeus Linn) and CHM 2 (Cuscuta chinensis Lam, Fructus Ligustri Lucidi, and Rubus idaeus Linn) induced recovery of the estrous cycle and the LH/FSH ratio, and reduction of androstenedione levels and the free androgen index (Gu et al., 2015). Gui Zhu Yi Kun formula (GZYKF) (Semen Cuscutae, Rhizoma Atractylodis Macrocephalae, Angelica sinensis, Adenophora tetraphylla, Plantago asiatica, Rubia cordifolia, and Luffa cylindrical) reduced mRNA expressions of Beclin 1, light chain 3, and tumor suppressor p53 (Xing et al., 2017). HemoHIM (Angelica Radix, Cnidii Rhizoma, and Paeonia Radix)-treated SD female rats showed increased number and size of corpora lutea and a decreased level of NGF protein in ovaries (Kim et al., 2009). Heqi san [Curculigo orchioides Gaertn., Schisandra chinensis (Turcz.) Baill., Cynanchum otophyllum C. K. Schneid., Citrus medica L. var. sarcodactylis Swingle, Crataegus pinnatifida Bunge, Rhus chinensis Mill., Clinopodium megalanthum (Diels) C. Y. Wu & Hsuan ex H. W. Li, Cuscuta chinensis Lam., Poncirus trifoliata (L.) Raf., Hordeum vulgare L., Polygala tenuifolia Willd., and Epimedium davidii Franch.] was found to reduce LH and testosterone levels, and to mitigate insulin resistance (Zhao et al., 2017). A novel herbal immunomodulator drug (IMOD) (Rosa canina, Urtica dioica, and Tanacetum vulgare)-treated female albino Wistar rats showed fewer incidences of ovarian atretic and cystic follicles, and a higher number of corpora lutea (Rezvanfar et al., 2012). Kyung-Ok-Ko (KOK) [Rehmannia glutinosa Liboschitz var. purpurae Makino (Scrophulariaceae), Lycium chinense Miller (Solanaceae), Aquillaria agallocha Roxburgh (Thymelaeaceae), Poria cocos Wolf (Polyporaceae), Panax ginseng C.A. Meyer (Araliaceae), and honey] administration induced the recovery of the estrous cycle and cluster of differentiation (CD)11b, CD3, IL-1β, IL-6, TNF-α, IL-8, monocyte chemoattractant protein (MCP)-1, and iNOS mRNA expression reduction in ovaries of female SD rats (Jang et al., 2014; Lee et al., 2016). Female SD rats treated with Xiao-Yao-San (XYS) (radix bupleuri, angelica, radix paeoniae alba, rhizoma atractylodis macrocephalae, poria, ginger, mint, and glycyrrhizae) showed a reduced number of cystic follicles and lowered terminal deoxynucleotidyl transferase dUTP nick end labeling (TUNEL) positive cells in the antral follicles (Sun et al., 2017). The data are summarized in **Table 3**.

**Table 3:** *In vitro* and *in vivo* studies of herbal formula decoctions (n=10).

Interventions	Components	Target cells or animal models	Herbal extraction	Route of administration	Dosage	Results	Author (year)
Bushen Tongmai Recipe	Astragalus membranaceus Bge, Radix Polygoni Multiflori, Herba Cistanches, Radix Salviae Miltiorrhizae, Radix Notoginseng, Radix Puerariae, Herba Epimedii, Rhizoma Chuanxiong, and Radix Rehmanniae	<*in vivo*>^(6)^ Female SD rats (aged 22 days)	not reported	Gastric perfusion	1.5 ml For 14 days	1) Morphology: stratum granulosum of ovarian follicles ↑ 2) FBG (-), serum fasting insulin ↓, insulin sensitive index ↑ 3) mRNA expressions of PKBα in the hepatic, adipose, and skeletal muscle tissues ↑, protein expressions of PKBα in the hepatic and adipose tissues ↑, protein expressions of PKBα in thecal and granulosa cells of antral follicles ↑	Li et al. (2010)
Changbudodam-Tang & Yongdamsagan-Tang	Rhizoma atractylodis, Rhizoma cyperi, Fructus ponciri, Pericarpium citri nobilis, Poria, Arisaematis Rhizoma, Radix glycyrrhizae, Massa Medicata Fermentata, and Zingiberis Rhizoma & Radix gentianae, Bupleuri Radix, Alimatis Rhizoma, Lignum akebiae, Plantaginis Semen, Hoelen, Rehmanniae Radix, Angelicae Gigantis Radix, Gardeniae Fructus, Scutellariae Radix, and Radix glycyrrhizae	<*in vivo*>^(3)^ Virgin adult cycling SD rats (190–210 g)	Solvent: 1,000 ml of boiling distilled water Time: 43 h	Oral (zonde needle)	50 mg/kg/2d for Changbudodam-Tang, & 40 mg/kg/2d for Yongdamsagan-Tang For 60 days	1) Morphology: numbers of corpora lutea and corpora albicantia ↑, number of cystic follicles ↓, number of growing secondary follicle ↑ 2) Expression of NGF in ovaries ↓, expression of NGF in pituitary and hippocampus (-) 3) Ovarian weight ↑, body weight (-)	Lee et al. (2003)
CHM1 & CHM2	Radix Astragali, Radix Rehmanniae Preparata, Cuscuta chinensis Lam, Fructus Ligustri Lucidi, Fructus Psoraleae, Radix Salviae Miltiorrhizae, Rubus idaeus Linn & Cuscuta chinensis Lam, Fructus Ligustri Lucidi, Rubus idaeus Linn	<*in vivo*>^(2)^ Neonatal female SD rats (aged 9 days)	Solvent: boiling water Time: twice for 60 min	Oral	0.54(CHM1), 0.24(CHM2) g/kg/d For 12 weeks	1) Recovery of estrous cycle 2) LH/FSH ↓, free androgen index ↓, androstenedione ↓ 3) mRNA expression of PPARG1 in ovaries ↑, mRNA expression of HDAC3 in ovaries ↓	Gu et al. (2015)
GZYKF	Semen Cuscutae, Rhizoma Atractylodis Macrocephalae, Angelica sinensis, Adenophora tetraphylla, Plantago asiatica, Rubia cordifolia, and Luffa cylindrica	<*in vitro*> Granulosa cells from female SD rats (aged 23–25 days, 60 ± 10 g)	Solvent: water Time: room temperature for 1 h and 100˚C for 1 h	–	13% rat serum containing GZYKF (administration of 5, 10, 20 g/kg/d for 4 days in low, medium, high-dose rat groups) For 72 h	1) mRNA expression of Beclin-1 ↓ 2) mRNA expression of LC3 ↓ (only from high-dose group) 3) protein expression of Beclin-1 ↓ 4) Protein expression of LC3 (-) 5) mRNA expression of tumor suppressor p53 ↓, mRNA expression of sestrin2 ↓(only from low and medium-dose groups) 6) mRNA expressions of TSC1, TSC2 and mTOR (-) 7) mRNA expression of AMPK ↑(only from high-dose group) 8) Protein expressions of mTOR, p-mTOR, tumor suppressor p53, AMPKα and sestrin ↑	Xing et al. (2017)
HemoHIM	Angelica Radix, Cnidii Rhizoma, and Paeonia Radix	<*in vivo*>^(3)^ SD female rats (210–230 g)	Solvent: boiling water Time: 4 h	[1] Oral [2] Intraperitoneally	[1] 100 mg/kg/d For 35 days [2] 50 mg/kg/2d For 35 days	1) Body weight (-), ovary weight ↑(only oral 100 mg/kg/d) 2) Morphology: number and size of corpora lutea ↑, cystically dilated atretic follicles ↑ 3) Level of NGF protein in ovaries ↓	Kim et al. (2009) Pak et al. (2009)
Heqi san	Curculigo orchioides Gaertn., Schisandra chinensis (Turcz.) Baill., Cynanchum otophyllum C. K. Schneid., Citrus medica L. var. sarcodactylis Swingle, Crataegus pinnatifida Bunge, Rhus chinensis Mill., Clinopodium megalanthum (Diels) C. Y. Wu & Hsuan ex H. W. Li, Cuscuta chinensis Lam., Poncirus trifoliata (L.) Raf., Hordeum vulgare L., Polygala tenuifolia Willd., and Epimedium davidii Franch.	<*in vivo*>^(1)^ Female SD rats (aged 3 months, 300 ± 20 g)	Solvent: ddH2O Time: twice for 1.5 h	Oral cannula	8.1 g/kg/d For 30 days	1) LH ↓, T ↓, E2 (-) 2) Insulin resistance ↓ 3) Histology: ovarian volume ↓, organ coefficient ↑, cystic dilatation in the ovarian follicles ↓, the oocytes in the follicles ↑, the number of granule cell layers ↑ 4) mRNA expressions of GLUT4 and PTEN in ovaries ↑ 5) Protein expressions of IRS-1 and PTEN in ovaries ↓ 6) Protein expressions of GLUT4 and p-IRS-1 in ovaries (-) 7) Expression of rno-miR-144-3p in ovaries ↓ 8) Expressions of rno-miR-30c-2-3p, rno-miR-486, and rno-miR-3586-3p in ovaries ↓ 10) Expression of rno-miR-146b-5p in ovaries ↑	Zhao et al. (2017)
IMOD	Rosa canina, Urtica dioica, and Tanacetum vulgare	<*in vivo*>^(4)^ Adult female albino Wistar rats (200 ± 10 g)	Solvent: ethanol (96%) Time: 30 days	Intraperitoneally	30 mg/kg/d For 21 days	1) Body weight ↓, ovarian weight ↓ 2) Recovery of estrous cycle 3) Histomorphology: total populations of ovarian atretic and cystic follicles ↓, number of corpora lutea ↑ 4) Serum levels of lipid peroxidation, peroxynitrite, TNF-α, and T ↓; serum levels of SOD, catalase, GPx, P, and E2 ↑ 5) Ovarian levels of lipid peroxidation, peroxynitrite, PGE ↓; ovarian levels of SOD, catalase, and GPx ↑	Rezvanfar et al. (2012)
Kyung-Ok-Ko	Rehmannia glutinosa Liboschitz var. purpurae Makino (Scrophulariaceae), Lycium chinense Miller (Solanaceae), Aquillaria agallocha Roxburgh (Thymelaeaceae), Poria cocos Wolf (Polyporaceae), Panax ginseng C.A. Meyer (Araliaceae), and honey	<*in vivo*>^(1)^ Female SD rats (aged 23 days)	Solvent: hot water (80˚C) Time: 72 h	Oral	0.5, 1.0, 2.0 g/kg/d For 40 days	1) Body weight ↓ 2) Ovary weight ↓(only 2.0 g/kg/d) 3) Number of follicular cysts ↓(only 2.0 g/kg/d), size of follicular cysts ↓(only 2.0 g/kg/d) 4) Insulin (-), insulin resistance (-) 5) E2 ↓(only 2.0 g/kg/d), P (-) 6) Recovery of estrous cycle (only 2.0 g/kg/d) 7) CD8 (+) in lymph node ↓(only 2.0 g/kg/d), CD4 (+) in lymph node (-) 8) CD8 (+) in ovary ↓(only 2.0 g/kg/d) 9) mRNA expressions of CD11b and CD3 in ovaries ↓(only 2.0 g/kg/d), Iba-1 immunoreactivity ↓(only 2.0 g/kg/d) 10) mRNA expressions of IL-1β, IL-6, TNF-α, IL-8, MCP-1, and iNOS in the ovaries ↓(only 2.0 g/kg/d) 11) mRNA expressions of EGF and TGF-β in the ovaries ↑(only 2.0 g/kg/d)	Jang et al. (2014)
	Rehmannia glutinosa, Liboschitz var. purpurae, Lycium chinense, Aquillaria agallocha, Poria cocos, Panax ginseng, and honey	<*in vivo*>^(1)^ Female SD rats (aged 23 days)	not reported	Oral	2.0 g/kg/d For 20 days	1) Body weight ↓ 2) Histomorphometric study: size and number of follicular cysts in the ovaries ↓, sizes of uteri ↓, outer diameters of uteri ↓, normalization of endometrium, myometrium, epimetrium, and cystic glands 3) Recovery of estrous cycle 4) Uterine weight ↓ 5) TUNEL positive cells ↓ 6) Iba-1+ macrophages ↓, CD4+ T cells ↓, CD8+ T cells ↓ 7) mRNA expressions of IL-1β, IL-6, IL-8, and MMP-3 in uteri ↓ 8) mRNA expressions of IGF-β, TGF-β, TGF-β1, and VEGF in uteri ↑	Lee et al. (2016)
XYS	Radix Bupleuri, Angelica, Radix Paeoniae Alba, Rhizoma Atractylodis Macrocephalae, Poria, Ginger, Mint, and Glycyrrhizae	<*in vivo*>^(7)^ [1] Female SD rats (aged 8 weeks, 220 ± 20 g) <*in vitro*> [2] Granulosa cells from female SD rats (aged 21 days)	not reported	Gavage	[1] 0.505, 1.01 g/kg/d For 4 weeks [2] 15% rat serum containing XYS (administration of 1.01 g/kg/d for 3 days in female SD rats) For 24 h	[1] 1) Recovery of estrous cycle 2) Morphology: number of cystic follicles ↓, recovery of cystic follicles formation and follicle development abnormalities 3) Noradrenaline in serum ↓(only 0.505 g/kg), noradrenaline in ovary ↑, LH ↓(only 1.01 g/kg), E2 (-), P ↑(only 1.01 g/kg) 4) Expression of β2R in the primordial and primary follicles ↓ 5) TUNEL positive cells in the antral follicles ↓ 6) Bax/Bcl-2 (-), cleaved caspase-3/GAPDH ↓(only 1.01 g/kg) 7) Expression of LC3A in the granulosa cells of the antral and cystic follicles ↓ 8) Conversion of LC3A-I to LC3A-II ↓(only 1.01 g/kg), conversion of LC3BI to LC3B-II ↓ 9) Phosphorylation of S6K I and Akt in ovarian tissues ↑ 10) Expression of DβH and c-fos in locus coeruleus ↓(only 1.01 g/kg) [2] 1) Expression of β2R ↓ 2) Expression of LC3 ↓(only 1.01 g/kg) 3) Conversion of LC3A-I to LC3A-II ↓(only 1.01 g/kg), conversion of LC3BI to LC3B-II ↓(only 1.01 g/kg) 4) Phosphorylation of S6K I and Akt ↑(only 1.01 g/kg)	Sun et al. (2017)

Akt, protein kinase B; AMPK, Adenosine monophosphate-activated protein kinase; Bax, B-cell lymphoma-2 associated X protein; Bcl-2, B-cell lymphoma-2; CD, Cluster of differentiation; CHM, Chinese herbal medicine; Cleaved caspase-3, Cleaved cysteinly aspartate specific proteinase-3; DβH, Dopamine beta hydroxylase; EGF, Epidermal growth factor; FBG, Fasting blood glucose; FSH, Follicle-stimulating hormone; GAPDH, Glyceraldehyde 3-phosphate dehydrogenase; GPx, Glutathione peroxidase; GULT4, Glucose transporter type 4; GZYKF, Gui Zhu Yi Kun formula; HDAC, Histone deacetylase; Iba1, Ionized calcium-binding adapter molecule 1; IL, Interleukin; iNOS, Inducible nitric oxide synthase; IRS, Insulin receptor substrate; LC, Light chain; LH, Luteinising hormone; MCP-1, Monocyte chemoattractant protein-1; mTOR, Mammalian target of rapamycin; NGF, Nerve growth factor; PGE, Prostaglandin E; PKBα, Protein kinase Bα; PPARG, Peroxisome proliferator-activated receptor gamma; PTEN, Phosphatase and tensin homolog; S6K I, Ribosomal protein S6 kinase polypeptide I; SD, Sprague-Dawley; SOD, Superoxide dismutase; TNF, Tumor necrosis factor; TSC, Tuberous sclerosis protein; TUNEL, Terminal deoxynucleotidyl transferase dUTP nick end labeling; VEGF, Vascular endothelial growth factor; XYS, Xiao-Yao-San; β2R, Beta 2 adrenergic receptor.

In vivo polycystic ovary model by using ^(1)^ dehydroepiandrosterone; ^(2)^ testosterone propionate; ^(3)^ estradiol valerate; ^(4)^ letrozole; ^(5)^ dihydrotestosterone; ^(6)^ sodium prasterone sulfate; and ^(7)^ chronic unpredictable mild stress.

### The Actions of Herbal Medicines for Treating PCOS

Herbal medicines were found to normalize female hormones, (Zangeneh et al., 2010; Rezvanfar et al., 2012; Jang et al., 2014; Yu et al., 2014; Gu et al., 2015; Zhou et al., 2016; Atashpour et al., 2017; Sun et al., 2017; Xia et al., 2017; Zhao et al., 2017; Jelodar et al., 2018), diminish male hormones (Yang et al., 2011; Rezvanfar et al., 2012; Yu et al., 2014; Gu et al., 2015; Zhou et al., 2016; Xia et al., 2017; Zhao et al., 2017; Jelodar et al., 2018), recover the estrous cycle (Mohamadin et al., 2010; Yang et al., 2011; Rezvanfar et al., 2012; Jang et al., 2014; Soumya et al., 2014; Yu et al., 2014; Gu et al., 2015; Lee et al., 2016; Zhou et al., 2016; Sun et al., 2017; Wang et al., 2017; Xia et al., 2017), ameliorate insulin resistance (Li et al., 2010; Mannerås et al., 2010; Yang et al., 2011; Desai et al., 2012; Wang et al., 2017; Zhao et al., 2017), and improve lipid metabolism in PCOS model (Mannerås et al., 2010; Desai et al., 2012; Soumya et al., 2014). The actions of herbal medicines and relevant outcomes are shown in **Table 4**.

**Table 4:** The actions of herbal medicines for treating PCOS.

**Actions**	**Interventions**	**Outcomes**	**Author (year)**
**Normalizing female hormone**	<*in vitro*, *in vivo*> Atractylodes macrocephala koidz	LH ↓, FSH ↑, LH/FSH ↓ AMH ↓	Zhou et al. (2016)
	<*in vivo*> Chamomile	E2, LH, FSH ↓	Zangeneh et al. (2010)
	<*in vivo*> CHM1 & CHM2	LH/FSH ↓	Gu et al. (2015)
	<*in vivo*> Cryptotanshinone	LH ↓, FSH ↑, LH/FSH ratio ↓, P (-), E2 ↓	Xia et al. (2017)
		E2 (-), LH ↓, FSH (-), LH/FSH ↓	Yu Jang et al. (2014)
	<*in vivo*> Flaxseed	P ↑, E2 (-)	Jelodar et al. (2018)
	<*in vivo*> Ginger	LH ↓, FSH ↑, E ↓, P ↑	Atashpour et al. (2017)
	<*in vivo*> Heqi san	LH ↓, E2 (-)	Zhao et al. (2017)
	<*in vivo*> IMOD	P ↑, E2 ↑	Rezvanfar et al. (2012)
	<*in vivo*> Kyung-Ok-Ko	E2 ↓, P (-)	Jang Jang et al. (2014)
	<*in vitro*, *in vivo*> XYS	LH ↓, E2 (-), P ↑	Sun et al. (2017)
**Diminishing male hormone**	<*in vitro*, *in vivo*> Atractylodes macrocephala koidz	T ↓, SHBG (-), free androgen index ↓, androstenedione ↓,	Zhou et al. (2016)
	<*in vivo*> CHM1 & CHM2	Free androgen index ↓, androstenedione ↓	Gu et al. (2015)
	<*in vivo*> Cryptotanshinone	T ↓	Xia et al. (2017)
		T ↓, androstenedione ↓, SHBG ↑	Jang et al. (2014)
		Androstenedione ↓, T ↓	Yang et al. (2011)
	<*in vivo*> Flaxseed	T ↓, Dehydroepiandrosterone (-)	Jelodar et al. (2018)
	<*in vivo*> Heqi san	T ↓	Zhao et al. (2017)
	<*in vivo*> IMOD	T ↓	Rezvanfar et al. (2012)
**Recovery of the estrous cycle**	<*in vitro*, *in vivo*> Aloe vera gel	Recovery of estrous cycle	Maharjan et al. (2010)
	<*in vitro*, *in vivo*> Atractylodes macrocephala koidz	Recovery of estrous cycle	Zhou et al. (2016)
	<*in vivo*> CHM1 & CHM2	Recovery of estrous cycle	Gu et al. (2015)
	<*in vivo*> Cocus nucifera flower	Recovery of estrous cycle	Soumya et al. (2014)
	<*in vivo*> Cryptotanshinone	Normalization of estrous cycle	Xia et al. (2017)
		Recovery of estrous cycle	Yu et al. (2014) (Jang et al., 2014)
		Recovery of estrous cycle	Yang et al. (2011)
	<*in vivo*> IMOD	Recovery of the estrous cycle	Rezvanfar et al. (2012)
	<*in vivo*> Kyung-Ok-Ko	Recovery of estrous cycle	Lee et al. (2016)
	<*in vivo*> Kyung-Ok-Ko	Recovery of estrous cycle	Jang et al. (2014)
	<*in vivo*> Quercetin	Recovery of estrous cycle	Wang et al. (2017)
	<*in vitro*, *in vivo*> XYS	Recovery of estrous cycle	Sun et al. (2017)
**Ameliorating insulin resistance**	<*in vivo*> Aloe vera gel	OGTT profile improvement	Desai et al. (2012)
	<*in vivo*> Bushen Tongmai Recipe	Fasting blood glucose (-) Serum fasting insulin ↓ Insulin sensitive index ↑	Li et al. (2010)
	<*in vivo*> Cryptotanshinone	OGTT profile improvement	Yang et al. (2011)
	<*in vivo*> Heqi san	Insulin resistance ↓	Zhao et al. (2017)
	<*in vivo*> Labisia pumila var. alata	Insulin sensitivity ↑ (glucose infusion rate ↑) Plasma glucose (-)	Mannerås et al. (2010)
	<*in vivo*> Quercetin	Insulin ↓, IAUC ↓, FBG ↓ Insulin resistance ↓	Wang et al. (2017)
**Lipid metabolism improvement**	<*in vivo*> Aloe vera gel	Plasma levels of TC ↓, TG ↓, HDL-C ↑, LDL-C ↓ Liver cholesterol ↓ Activity of HMG-CoA reductase ↓ Activity of LCAT (-)	Desai et al. (2012)
	<*in vivo*> Cocus nucifera flower	TC ↓, VLDL-C ↓, HDL-C ↑, TG ↓	Soumya et al. (2014)
	<*in vivo*> Labisia pumila var. alata	TC ↓, TG ↓, HDL-C (-), LDL-C (-) Resistin ↑, adiponectin (-), leptin (-) mRNA expression of leptin in mesenteric adipose tissue ↓ mRNA expression of resistin and adiponectin in mesenteric adipose tissue (-)	Mannerås et al. (2010)

AMH, Anti-Müllerian hormone; CHM, Chinese herbal medicine; E, Estrogen; E2, Estradiol; FBG, Fasting blood glucose; FSH, Follicle-stimulating hormone; HDL-C, High density lipoprotein cholesterol; HMG-CoA, β-Hydroxy β-methylglutaryl-CoA; IAUC, Area under the curve of blood insulin release; LCAT, Lecithin–cholesterol acyltransferase; LDL-C, Low density lipoprotein cholesterol; LH, Luteinising hormone; OGTT, Oral glucose tolerance test; P, Progesterone; SHBG, Sex hormone-binding globulin; TC, Total cholesterol; TG, Triglyceride; VLDL-C, Very low density cholesterol; XYS, Xiao-Yao-San.

### The Mechanisms of Action for PCOS

The mechanisms underlying the beneficial effects of herbal medicines on PCOS model were as follows: the alleviation of inflammation (Rezvanfar et al., 2012; Jang et al., 2014; Lee et al., 2016; Wang et al., 2017; Zuo et al., 2017) and/or oxidative stress, (Rezvanfar et al., 2012; Zuo et al., 2017), the inhibition of autophagy and/or apoptosis (Lee et al., 2016; Sun et al., 2017; Xing et al., 2017), and the reduction of the ovarian NGF (Lee et al., 2003; Pak et al., 2005; Kim et al., 2009; Pak et al., 2009; Jung et al., 2011). The mechanisms of action of each in PCOS model and relevant outcomes are shown in **Table 5**.

**Table 5:** The mechanisms of action of herbal medicines on PCOS.

Mechanisms	Interventions	Outcomes	Author (year)
**Anti-inflammation or immunomodulatory**	<*in vitro*> Iridoid (genipin, geniposide, and geniposidic acid)	1) mRNA expressions of IL-1β, IL-6, IL-10 and iNOS ↓ 2) Over-secretion of nitrite ↓ 3) MiR-15b expression ↓	Zuo et al. (2017)
	<*in vivo*> Quercetin	1) IL-1β ↓, IL-6 ↓, TNF-α ↓ 2) Phosphorylation of IRS-1 ↑ 3) Nuclear translocation of NF-κB P65 ↓ 4) mRNA expressions of p22phox, OX-LDL, and TLR-4 in ovaries ↓ 5) Protein expressions of p22phox, OX-LDL, and TLR-4 in ovaries ↓	Wang et al. (2017)
	<*in vivo*> Kyung-Ok-Ko	1) Iba-1+ macrophages ↓, CD4+ T cells ↓, CD8+ T cells ↓ 2) mRNA expressions of IL-1β, IL-6, IL-8, and MMP-3 in uteri ↓ 3) mRNA expressions of IGF-β, TGF-β, TGF-β1, and VEGF in uteri ↑	Lee et al. (2016)
	<*in vivo*> Kyung-Ok-Ko	1) CD8 (+) in lymph node ↓, CD4 (+) in lymph node (-) 2) CD8 (+) in ovary ↓ 3) mRNA expressions of CD11b and CD3 in ovaries ↓, Iba-1 immunoreactivity ↓ 4) mRNA expressions of IL-1β, IL-6, TNF-α, IL-8, MCP-1, and iNOS in ovaries ↓ 5) mRNA expressions of EGF and TGF-β in ovaries ↑	Jang et al. (2014)
	<*in vivo*> IMOD	1) Serum TNF-α ↓ 2) Ovarian PGE ↓	Rezvanfar et al. (2012)
**Anti-oxidative stress**	<*in vitro*> Iridoid (genipin, geniposide, and geniposidic acid)	1) Phosphorylation and degradation of IκB ↓ 2) NF-κB P65 ↑ 3) Nuclear entry of NF-κB P65 ↓ 4) Scavenge O2- 5) SOD activity ↓, HO-1 mRNA expression ↓, catalase activity ↑	Zuo et al. (2017)
	<*in vivo*> IMOD	Ovarian and serum SOD, catalase, GPx ↓	Rezvanfar et al. (2012)
**Autophagy or apoptosis inhibition**	<*in vitro*> GZYKF	1) mRNA expression of Beclin-1 ↓ 2) mRNA expression of LC3 ↓ 3) Protein expression of Beclin-1 ↓ 4) Protein expression of LC3 (-) 5) mRNA expressions of tumor suppressor p53 and sestrin2 ↓ 6) mRNA expressions of TSC1, TSC2 and mTOR (-) 7) mRNA expression of AMPK ↑ 8) Protein expressions of mTOR, p-mTOR, tumor suppressor p53, AMPKα and sestrin ↑	Xing et al. (2017)
	<*in vitro*, *in vivo*> XYS	<*in vivo*> 1) Expression of β2R in the primordial and primary follicles ↓ 2) TUNEL positive cells in the antral follicles ↓ 3) Bax/Bcl-2 (-), cleaved caspase-3/GAPDH ↓ 4) Expression of LC3A in the granulosa cells of the antral and cystic follicles ↓ 5) Conversion of LC3A-I to LC3A-II ↓, conversion of LC3BI to LC3B-II ↓ 6) Phosphorylation of S6K I and Akt in ovarian tissues ↑ 7) Expression of DβH and c-fos in locus coeruleus ↓ <*in vitro*> 1) Expression of β2R ↓ 2) Expression of LC3 ↓ 3) Conversion of LC3A-I to LC3A-II ↓, conversion of LC3BI to LC3B-II ↓ 4) Phosphorylation of S6K I and Akt ↑	Sun et al. (2017)
	<*in vivo*> Kyung-Ok-Ko	TUNEL positive cells ↓	Lee et al. (2016)
**Ovarian NGF reduction**	<*in vivo*> Korean red ginseng	1) mRNA expression of NGF in ovaries ↓ 2) Protein expression of NGF in ovaries ↓	Jung et al. (2011)
	<*in vivo*> Korean red ginseng	1) mRNA expression of NGF in ovaries ↓ 2) Protein expression of NGF in ovaries ↓	Pak et al. (2009)
	<*in vivo*> HemoHIM	Protein expression of NGF in ovaries ↓	Pak et al. (2009)
	<*in vivo*> Korean red ginseng total saponins	1) Protein expression of NGF in ovaries ↓ 2) Protein expression of NGF in pituitary and hippocampus (-)	Pak et al. (2005)
	<*in vivo*> Changbudodam-Tang & Yongdamsagan-Tang	1) Expression of NGF in ovaries ↓ 2) Expression of NGF in pituitary and hippocampus (-)	Lee et al. (2003)

Akt, protein kinase B; AMPK, Adenosine monophosphate-activated protein kinase; Bax, B-cell lymphoma-2 associated X protein; Bcl-2, B-cell lymphoma-2; CD, Cluster of differentiation; Cleaved caspase-3, Cleaved cysteinly aspartate specific proteinase-3; DβH, dopamine beta hydroxylase; GAPDH, Glyceraldehyde 3-phosphate dehydrogenase; GPx, Glutathione peroxidase; GZYKF, Gui Zhu Yi Kun formula; HO-1, Heme oxygenase-1; Iba1, Ionized calcium-binding adapter molecule 1; IL, Interleukin; iNOS, Inducible nitric oxide synthase; IRS, Insulin receptor substrate; LC, Light chain; MCP-1, Monocyte chemoattractant protein-1; MMP, Matrix metalloproteinase; mTOR, Mammalian target of rapamycin; NF-κB, Nuclear factor kappa B; NGF, Nerve growth factor; OX-LDL, Oxidized low-density lipoprotein; PGE, Prostaglandin E; S6K I, Ribosomal protein S6 kinase polypeptide I; SOD, Superoxide dismutase; TLR, Toll-like receptor; TNF, Tumor necrosis factor; TSC, Tuberous sclerosis protein; TUNEL, Terminal deoxynucleotidyl transferase dUTP nick end labeling; XYS, Xiao-Yao-San; β2R, Beta 2 adrenergic receptor.

## Discussion

Our review of the literature published up to June 30, 2018 summarized the findings of *in vitro* and *in vivo* studies on the efficacy of herbal medicines for the treatment of PCOS model. A total of four compounds isolated from herbs (6 studies), nine individual herbal extracts (11 studies), and nine herbal formula decoctions (10 studies) were found to have inhibitory effects on PCOS. According to the results reported, herbal medicines normalized female hormones, diminished male hormones, recovered the estrous cycle, ameliorated insulin resistance, and improved lipid metabolism. We found that the potential inhibitory activity of herbal medicines could influence different aspects of PCOS, with the beneficial effects of herbal medicines arising mainly through anti-inflammation, anti-oxidative stress, inhibition of autophagy or apoptosis, and ovarian NGF reduction.

### Herbal Medicines Can Inhibit the Inflammatory Conditions of PCOS

Recent studies have further explored the etiology and pathology of PCOS. Scientists found that in the microenvironment of patients with PCOS, mild chronic inflammation is a hallmark of the syndrome (Nestler, 2000). PCOS has been relevant in chronic inflammation (Benson et al., 2008), and macrophages have been its major pathogenesis (Wu et al., 2004). Ovarian macrophages produce cytokines, chemokines, and growth factors in both the normal and the inflammatory processes of the ovary. The macrophages can orchestrate apoptosis and tissue remodeling, which are involved in folliculogenesis, ovulation, and formation of the corpus luteum (Benson et al., 2008). Given the critical role of macrophages in PCOS (Benson et al., 2008), numerous studies have compared cytokine levels in serum and in follicular fluids in PCOS patients. TNF-α and IL-6 levels in serum and in follicular fluids were elevated in non-obese/non-diabetic PCOS patients treated with gonadotrophins (Amato et al., 2003). Macrophage inflammatory protein-1α and MCP-1 were increased in PCOS patients and associated with adiposity (Glintborg et al., 2009). iNOS, cyclooxygenase-2 (COX-2), and transforming growth factor (TGF)-β activity were increased in the ovaries of PCOS patients (Elia et al., 2006; Hatzirodos et al., 2011), and iNOS and COX-2 activity were prevented by metformin administration (Elia et al., 2006). These results suggest that the immune system is relevant to the pathogenesis of PCOS. Therefore new remedies targeting this inflammatory process can be a therapeutic alternative to the current treatment.

In this review, iridoids significantly inhibited IL-1β, IL-6, IL-10, and iNOS expression, thereby inhibiting inflammatory conditions (Zuo et al., 2017). Quercetin also significantly reduced the levels of IL-1β, IL-6, and TNF-α, and decreased nuclear translocation of nuclear factor kappa B (NF-κB) in an insulin-resistant PCOS rat model (Wang et al., 2017). Pre-administration of KOK diminished the increased expression of ionized calcium-binding adapter molecule -1 (+) macrophages in the theca cell layer of cysts and the stroma. KOK also increased mRNA expression of CD11b and CD3 in PCOS ovarian tissue. Pre-administration of KOK significantly decreased the increased levels of IL-1β, IL-6, IL-8, TNF-α, MCP-1, and iNOS; and increased the reduced mRNA expression of epidermal growth factor and TGF-β in PCOS ovaries. These results demonstrated that KOK regulates the expression of inflammatory mediators in the dehydroepiandrosterone (DHEA)-induced PCOS model (Jang et al., 2014). Inflammatory mediators were also regulated in the endometrium of the uterus by KOK administration, which has been shown to prevent endometrial hyperplasia in PCOS models (Lee et al., 2016). It was also shown that TNF-α in serum and prostaglandin E (PGE) in the ovary were decreased by IMODs (Rezvanfar et al., 2012).

### Herbal Medicines Can Attenuate Oxidative Stress in PCOS

Reactive oxygen species are important signal molecules in the regulation of physiological functions in female reproduction, including steroidogenesis, folliculogenesis, oocyte maturation, corpus luteum function, and luteolysis (Agarwal et al., 2005). In addition, they play a key role in the pathological processes of female reproduction (Agarwal et al., 2003; Agarwal et al., 2008). Oxidative stress is a condition in which the equilibrium between antioxidant capacity of the body and toxic oxygen- and/or nitrogen-derived products is impaired. Consequently, free radicals are insufficiently detoxified by cellular antioxidants. Oxidative stress plays an important role in the female reproduction (Ruder et al., 2008; Ruder et al., 2009; Vakilian et al., 2009), and there is increasing literature on the effects of increased oxidative stress markers in infertile females, and on their involvement in the pathophysiology of PCOS (Joo et al., 2010; Mohamadin et al., 2010). PCOS is characterized by chronic inflammation, oxidative stress, and abnormal microRNA expression (Zhao et al., 2015; Zuo et al., 2016). Since it is known that inflammation and oxidative stress are closely linked, elevated oxidative stress usually results from, and leads to, an inflammatory condition (Zuo et al., 2016). It is difficult to separate inflammation from oxidative stress, and it has been proposed in recent years that inflammation and oxidative stress comprise the main foundation of disease occurrence (Duleba and Dokras, 2012).

In this review, IMODs (Rezvanfar et al., 2012) and iridoids (Zuo et al., 2017) showed positive effects on oxidative/nitrosative stress, either directly or indirectly, mainly by reducing free radicals and inhibiting inflammatory cytokines in PCOS rats. Administration of IMODs significantly reduced lipid peroxidation (a marker of oxidative stress) and increased superoxide dismutase, catalase and glutathione peroxidase (markers of antioxidant potential) levels in hyperandrogenism-induced PCOS. In addition, peroxynitrite (a marker of nitrosative stress), TNF-α, and PGE levels were significantly reduced by IMODs. Furthermore, these effects of IMODs were consistent with histologic evidence, which showed significant improvement in the microscopic characteristics of folliculogenesis compared with those in the control group (Rezvanfar et al., 2012). The NF-κB signaling system is known as a dominant paradigm for specific signal transduction molecules, gene activation, and regulatory proteins in response to inflammation (Ivanenkov et al., 2011). The iridoids efficiently attenuated the lipopolysaccharide induced elevation of IκB phosphorylation levels, decreased IκB expression levels, and decreased NF-κB P65, indicating that the iridoids exert their antioxidant effects *via* the NF-κB pathway (Zuo et al., 2017).

### Herbal Medicines Can Regulate Apoptosis and/or Autophagy in PCOS

The survival or death of granulosa cells is recognized as a critical factor impacting the fate of follicles (Matsuda et al., 2012). Apoptosis and autophagy are two forms of programmed cell death. Autophagy is the process by which an autophagosome, which is a double-membrane vesicle, carries cytoplasmic material to the lysosome (Mizushima and Komatsu, 2011). It has been reported that both apoptosis and autophagy can be induced in granulosa cells, and that they are involved in the control of follicular development (Choi et al., 2010; Choi et al., 2013). Granulosa cells are recognized as critical players in follicle development. They produce estradiol, insulin-like growth factors, and other cytokines in the ovary and express the receptors for estradiol, LH, and FSH, (Juengel et al., 2006), all of which participate in the regulation of follicle development. Thus, any impairment of the granulosa cells may results in disordered development of follicles.

In the last decade, autophagy-related signaling pathways and their major protein regulators have been identified. The rat microtubule-associated protein 1 light chain 3 is associated with autophagosome membrane processing (Kabeya et al., 2000). Beclin-1 has also been demonstrated to have a critical role in autophagosome formation (Von Hoven et al., 2012). Furthermore, previous studies have identified tumor suppressor p53 (p53) as a dual modulator of autophagy in regulating cell death or survival (Vousden and Ryan, 2009; Zhang et al., 2010). At low energy levels, adenosine monophosphate-activated protein kinase (AMPK), which is activated by p53, is able to activate tuberous sclerosis complex 2, and therefore inhibit the mammalian target of rapamycin (mTOR) activity and increase autophagy (Feng et al., 2007). In this review, p53, which is activated by GZYKF in the nucleus, in turn activated AMPK and sestrin, acting as a feedback in mTOR inhibition, thereby activating autophagy. Autophagy is also modulated by phosphoinositide 3-kinase/protein kinase B (PI3K/Akt) signaling pathways (Pyo et al., 2012). In this review, XYS alleviated the reduction of phosphorylation of ribosomal protein S6 kinase polypeptide I and Akt, as well as the increase of microtubule-associated protein light chain 3-I to microtubule-associated protein light chain 3-II conversion both *in vivo* and *in vitro* (Sun et al., 2017).

The endometria of most PCOS patients are thick and exhibit simple, complicated, or atypical hyperplasia or malignant transformation that may be correlated with endometrial cell apoptosis (Villavicencio et al., 2007). Hyperandrogenism induced by DHEA is associated with a greater number of apoptotic cells in the endometria, and metformin (an insulinomimetic or insulin-sensitizing agent) is able to reduce the increased number of apoptotic cells (Elia et al., 2009). In this review, apoptotic cell death was evaluated by TUNEL staining. Apoptotic cells were rarely detected by TUNEL staining in the uterine tissue of the sham or the KOK-alone group. However, the number of TUNEL-positive cells was increased in the endometria of DHEA group. The increased number of apoptotic cells was significantly reduced after administration of KOK. These results indicate that pre-administration of KOK inhibited DHEA-induced endometrial malformation by reducing endometrial apoptosis (Lee et al., 2016).

### Herbal Medicines Can Reduce the Level of NGF in PCOS

Previous studies have shown that PCOS is associated with abnormal activation of the sympathetic nervous system, resulting in increased catecholaminergic nerves (Semenova, 1969), impaired norepinephrine metabolism (Garcia-Rudaz et al., 1998), and increased activity of sympathetic nerves *via* the superior ovarian nerve (Lara et al., 1993). The development and function of ovarian sympathetic innervation depend on the ovary for the production of NGF, a target-derived neurotrophin required for peripheral sympathetic system development (Levi-Montalcini, 1987). The neurotrophin family, implicates the NGF receptor and NGF mRNA in ovulation and in the pathophysiology of PCOS (Lara et al., 2000; Stener-Victorin et al., 2003). In rat ovaries, NGF is principally synthesized in the cells of the follicular wall (Dissen et al., 1996), and in PCOS, the activation of NGF may be a factor involved in enhancing norepinephrine outflow to the gland, which is induced by estradiol valerate (EV) (Lara et al., 2000) PCOS exhibits a high intraovarian nerve fiber density that is associated with sympathetic hyperresponsiveness (Stener-Victorin et al., 2003).

In this review, the administration of the herbal formulas Changbudodam-Tang and Yongdamsagan-Tang significantly decreased elevated NGF in the ovaries with little effect on brain tissue (Lee et al., 2003). HemoHIM also normalized NGF, lowered the high number of antral follicles, and increased the number of corpora lutea in PCOS. These results are consistent with those of previous studies on the beneficial effects of HemoHIM in the prevention and treatment of PCOS (Pak et al., 2009). In addition, administration of Korean red ginseng extract (Pak et al., 2009; Jung et al., 2011) and Korean red ginseng total saponins (Pak et al., 2005) significantly decreased the expression of NGF protein and NGF mRNA, compared with those in EV-treated ovaries.

This study reviewed the evidence for herbal medicines that may be used to treat PCOS and its associated symptoms, and the findings are intended to add to clinicians’ understanding of the mechanisms of action of herbal medicines in PCOS treatment. The main limitation of our study is the heterogeneity of interventions. Furthermore, our study did not perform a quality assessment of each study or a quantitative synthesis of the outcomes. Further studies that include methodological quality assessment and quantitative synthesis of outcomes are warranted.

## Conclusion

In this review, a total of 27 studies involving 22 herbal medicines exhibited beneficial effects on PCOS. Herbal interventions in the 27 studies comprised four compounds isolated from herbs (6 studies), nine individual herbal extracts (11 studies), and nine herbal formula decoctions (10 studies). Herbal medicines were shown to normalize female hormones, diminish male hormones, recover the estrous cycle, ameliorate insulin resistance, and improve lipid metabolism in PCOS. The mechanisms underlying the beneficial effects of herbal medicines on PCOS are associated with anti-inflammation, anti-oxidative stress, inhibition of autophagy and/or apoptosis, and ovarian NGF reduction. Herbal medicines can be considered as promising resources in the development of effective therapeutic agents for PCOS.

## Author Contributions

I-HC and KP designed the study. C-YK and KP searched the articles and analyzed the data. KP wrote the manuscript and I-HC revised it. All authors contributed to the article and approved the submitted version.

## Funding

This work was supported by National Research Foundation of Korea (NRF) grant funded by the Korea government (MSIP) (No.NRF-2017R1C1B1006387) and the Korean Society of Ginseng (2019).

## Conflict of Interest

The authors declare that the research was conducted in the absence of any commercial or financial relationships that could be construed as a potential conflict of interest.

## Abbreviations

Akt, protein kinase B; AMH, Anti-Müllerian hormone; AMPK, adenosine monophosphate-activated protein kinase; CD, cluster of differentiation; CHM, Chinese herbal medicine; COX, cyclooxygenase; DHEA, dehydroepiandrosterone; EV, estradiol valerate; FSH, Follicle-stimulating hormone; GZYKF, Gui Zhu Yi Kun formula; HDL-C, High density lipoprotein cholesterol; IL, interleukin; IMOD, immunomodulator drug; iNOS, inducible nitric oxide synthase; KOK, Kyung-Ok-Ko; LDL-C, Low density lipoprotein cholesterol; LH, Luteinising hormone; MCP, monocyte chemoattractant protein; MeSH, medical subject heading; mTOR, inhibit mammalian target of rapamycin; NF-κb, nuclear factor kappa B; NGF, nerve growth factor; p53, tumor suppressor p53; PCOS, polycystic ovary syndrome; PGE, prostaglandin E; PI3K, phosphoinositide3-kinase; SD, Sprague Dawley; SHBG, sex hormone binding globulin; TC, Total cholesterol; TG, Triglyceride; TGF, transforming growth factor; TNF, tumor necrosis factor; TUNEL, terminal deoxynucleotidyl transferase dUTP nick end labeling; VLDL-C, Very low density cholesterol; XYS, Xiao-Yao-San.
